# Childhood Famine Exposure and Late‐Life Edentulism: Evidence From Natural Experiments

**DOI:** 10.1111/jcpe.70190

**Published:** 2026-08-16

**Authors:** Sebastian‐Edgar Baumeister, Colman McGrath, Thomas Kocher, Gustavo G. Nascimento

**Affiliations:** ^1^ Institute of Health Services Research in Dentistry, University of Münster Münster Germany; ^2^ Applied Oral Sciences and Community Dental Care Faculty of Dentistry, the University of Hong Kong Pokfulam Hong Kong S.A.R. China; ^3^ Department of Restorative Dentistry, Periodontology, and Endodontology University Medicine Greifswald Greifswald Germany; ^4^ School of Dentistry University of Utah Salt Lake City Utah USA

**Keywords:** childhood undernutrition, developmental origins of health and disease, edentulism, famine, natural experiment

## Abstract

**Aim:**

To examine whether childhood famine exposure affects late‐life edentulism using natural experiments from Asia, Europe and America.

**Materials and Methods:**

We utilised historical famines as exogenous shocks. Data were pooled from 10 population‐based studies across eight countries (165,586 participants). Because the famine‐exposed cohort is older at survey than the comparison cohort, we separated famine exposure from the birth‐cohort age gradient using a regression discontinuity in time, an interrupted time series, a region‐of‐birth famine‐intensity difference‐in‐differences, a pre‐famine comparison cohort and applied negative controls.

**Results:**

The crude positive association (risk ratio [RR] 1.69) was eliminated after adjustment for current age (RR 1.07, 95% confidence interval (CI) 0.92–1.23), and the famine cohort did not differ from the older pre‐famine cohort (RR 0.97). The regression discontinuity (+0.13% points, 95% CI: −1.50 to +1.77), interrupted time series (−4.01% points, *p* = 0.15) and the region‐of‐birth difference‐in‐differences in the two Chinese cohorts (unadjusted, both *p* ≥ 0.07) were null, as were all negative controls.

**Conclusions:**

Once the birth‐cohort age gradient is accounted for, childhood famine exposure shows no detectable effect on late‐life edentulism; the crude positive association is largely explained by age confounding, which cross‐sectional famine‐cohort comparisons do not address.

## 1 Introduction

The global burden of childhood undernutrition has declined over recent decades, yet progress remains fragile, with a substantial risk of resurgence and persistence of childhood malnutrition driven by climate change and conflict in settings with vulnerable food systems. Concurrently, acute disruptions in food systems have driven global food prices to 116% above 2000 levels, exacerbated by the COVID‐19 pandemic and the Russia–Ukraine war (Landin Basterra et al. 2025). These disruptions do not merely represent transient economic hardships but also pose a generational threat to public health. Nutritional deprivation during the formative years of life leaves a biological imprint that may manifest as chronic disease decades later, burdening healthcare systems and diminishing the quality of life for ageing populations.

The first years after conception constitute a critical window of developmental plasticity during which undernutrition can negatively affect adult health (Gluckman et al. 2008). This concept is central to the Developmental Origins of Health and Disease (DOHaD) hypothesis, which posits that environmental exposures during sensitive periods of development induce permanent changes in tissue structure and function. Adverse nutritional conditions in utero are associated with increased risks of cardiovascular disease, stroke and diabetes mellitus in old age (Grey et al. 2021; Li et al. 2024; Li and Lumey 2017, 2022). The ‘thrifty phenotype’ hypothesis suggests that a foetus or young child deprived of nutrition adapts metabolically to survive in a resource‐poor environment; however, this adaptation becomes maladaptive if the individual is later exposed to an obesogenic or nutrient‐rich environment, leading to metabolic dysregulation.

While convincing evidence for the DOHaD hypothesis links prenatal malnutrition with an increased risk of non‐communicable disease risk later in life, less is known about the long‐term sequelae of malnutrition during postnatal periods of developmental plasticity such as childhood (Grey et al. 2021). Extensive evidence supports the impact of exposure to malnutrition during childhood on the long‐term increased risk of cardiometabolic diseases. The evidence is strongest for cardiovascular disease and diabetes mellitus, suggesting that the window of vulnerability extends well beyond birth (Grey et al. 2021). During childhood, rapid somatic growth, organ maturation and immune system calibration require substantial energy and micronutrient inputs. Deficits during these periods may compromise immune function and inflammatory regulation pathways that plausibly affect oral tissues as well as systemic health. Despite this evidence, the oral‐health consequences of childhood undernutrition remain poorly characterised. Edentulism is a debilitating condition that serves as a marker of cumulative oral disease (Aida 2021; Elani et al. 2017; Hunter et al. 2024; Matsuyama et al. 2019). It compromises nutritional intake, speech and psychosocial well‐being, yet its early‐life nutritional determinants are largely unknown.

To address this gap, we leveraged historical famines and hunger periods as natural experiments to estimate the effect of early‐life famine exposure on late‐life edentulism. A recent cross‐sectional analysis of the 1959–1961 Chinese Great Famine reported higher adjusted odds of adult edentulism among the exposed cohort (Gu et al. 2026). Because famine exposure is fixed at birth, however, the exposed and unexposed cohorts occupy near‐non‐overlapping age bands, so adjusting for age as a covariate incompletely separates exposure from the steep age gradient in edentulism and leaves residual age confounding. We extend this literature to 10 famines across Asia, Europe and the Americas, and use quasi‐experimental designs—regression discontinuity in time, interrupted time series and a region‐of‐birth famine‐intensity difference‐in‐differences—that separate famine exposure from the birth‐cohort age gradient.

## 2 Materials and Methods

### 2.1 Natural Experiments and Data

This study leveraged several famines and hunger precipitated by war or drought after 1900 in Europe, Asia and America as natural experiments. Table 1 provides a description of the historical contexts of these natural experiments and the data used in the analyses. From a harmonised pool of 205,768 records across the 10 studies, the analytical sample comprised 165,586 participants with complete data on edentulism, birth year and sex, with study‐specific sample sizes ranging from 4115 to 73,179 (Table S1). The survey wave analysed for each study is given in Table 1.

**TABLE 1:** Context of natural experiments and data used for identification of effects of famine and undernutrition on edentulism.

Context (country)	Famine or war period details	Data source	Study description and analytical sample
China	Time Period: 1942–1943 (Henan) & 1959–1961 (Great Famine)	CLHLS (2008) & CHARLS (2011/2012)	CLHLS: Retrospective data on childhood starvation.
	Spread: National (1959–1961), devastating in rural areas due to weather and policy.		CHARLS: National sample of residents aged 45+. Includes childhood health history.
	Energy Intake: 1500–1700 kcal/day (1960 trough); below 1000 kcal in severely hit regions.		
Russia	Time Period: 1946–1947 (Post‐WWII famine)	SAGE—Russia (Wave 1)	Part of the multi‐country WHO SAGE. Multi‐stage cluster sampling.
	Spread: Broadly affected the Soviet Union, particularly Ukrainian SSR, Russian SFSR, Byelorussian SSR, Moldavian SSR		
	Energy Intake: Urban rations dropped to ~1000–1200 kcal/day; rural areas often lower.		
India	Time Period: 1943–1944 (Bengal Famine)	LASI (Wave 1) & SAGE—India	LASI: Full‐scale national survey of 72,000+ individuals. SAGE: Longitudinal study focusing on the health and well‐being of older adults.
	Spread: Primarily Bengal region, with socio‐economic ripple effects across the subcontinent.		
	Energy Intake: Critical shortages; caloric intake for labouring classes fell below 1000 kcal/day.		
Netherlands	Time Period: 1944–1945 (Hongerwinter)	SHARE—Netherlands (Wave 3/SHARELIFE)	Retrospective life history wave (2008–2009). Ethics: Max Planck Society.
	Spread: Western Netherlands during German occupation and Allied advance.		
	Energy Intake: Dropped severely to 400–800 kcal/day during the winter of 1944.		
Spain	Time Period: 1939–1942 (Años del Hambre)	SHARE—Spain (Wave 3/SHARELIFE)	Retrospective life history interviews. Analysed cohort exposure relative to the Civil War and subsequent famine years.
	Spread: National, following the Spanish Civil War; exacerbated by international isolation.		
	Energy Intake: Official rations provided roughly 1000–1500 kcal/day, often unfulfilled.		
Germany	Time Period: 1945–1948 (Post‐WWII occupation)	SHARE—Germany (Wave 3/SHARELIFE)	Focuses on the ‘Hunger Years’ post‐capitulation. Sample restricted to those with valid childhood residence and health data.
	Spread: All occupation zones; urban centres heavily affected by infrastructure collapse.		
	Energy Intake: Rations varied by zone, often fluctuating between 1000 and 1250 kcal/day.		
Austria	Time Period: 1945–1948 (Post‐WWII occupation)	SHARE—Austria (Wave 3/SHARELIFE)	Focuses on the ‘Hunger Years’ post‐capitulation. Sample restricted to those with valid childhood residence and health data.
	Spread: Similar to Germany; Vienna and industrial areas suffered the most acute shortages.		
	Energy Intake: Average daily intake fell below 1200 kcal for significant portions of the urban population.		
Mexico	Time Period: 1914–1917 (Revolutionary Famine)	SAGE—Mexico (Wave 1)	Captures the oldest‐old survivors of the Mexican Revolution.
	Spread: ‘The Year of Hunger’ (1915); widespread in Mexico City and Zapatista‐controlled regions.		
	Energy Intake: Acute scarcity; extreme price inflation led to widespread starvation in urban centres.		

*Note:* Survey waves: CHARLS Wave 2 (2013–2014); CLHLS pooled cross‐sections (2008–2018); LASI Wave 1 (2017–2018); SAGE Wave 0 (2003); SHARE Wave 5 (2013).

Abbreviations: CHARLS, China Health and Retirement Longitudinal Study; CLHLS, Chinese Longitudinal Healthy Longevity Survey; LASI, Longitudinal Ageing Study in India; SAGE, Study on Global Ageing and Adult Health; SHARE, Survey of Health, Ageing and Retirement in Europe.

### 2.2 Outcome Variable

In the Chinese Longitudinal Healthy Longevity Survey (CLHLS), participants reported the number of remaining natural teeth (range: 0–32). As third molars are likely to be retained in China if they are disease‐free and well‐aligned (Chan et al. 2025), edentulism in this cohort was defined as the loss of ≥ 30 teeth. In all other datasets, participants were asked a binary question regarding whether they had lost all teeth.

### 2.3 Famine‐Cohort Exposure and Comparison Cohorts

The famine‐exposed cohort comprised participants aged 1–5 years during the famine years; comparison cohorts were participants aged 1–5 years in the immediate post‐famine period and participants aged 1–5 years in an equally long pre‐famine period. Cohort assignment was based on documented start and end years of each event by country; country‐specific definitions are detailed in Table 1.

### 2.4 Covariates

The design‐based analyses (regression discontinuity, interrupted time series and the region‐of‐birth difference‐in‐differences) do not rely on covariate adjustment, and the difference‐in‐differences is reported with and without covariates. We adjusted only the reduced‐form analysis for age, sex and early‐life socio‐economic position (SEP), the latter harmonised from the father's education or occupation. The SEP variable has no within‐study variation in the SAGE cohorts (all participants fall in the missing category), so it provides only between‐study adjustment there; study fixed effects absorb this between‐study heterogeneity.

### 2.5 Statistical Analyses

Because famine exposure is fixed at birth, the exposed and comparison cohorts occupy near‐non‐overlapping age bands, so adjusting for age as a covariate relies on extrapolation across those bands and incompletely separates exposure from the age gradient, leaving residual, functional‐form‐dependent age confounding. We therefore applied designs that separate the two. Our primary design was a regression discontinuity in time (RDiT; Hausman and Rapson 2018), with the running variable defined as birth year relative to the famine cohort cut‐off. We used bias‐corrected local‐polynomial inference (Calonico et al. 2014), honest bias‐aware confidence intervals for the discrete (integer birth‐year) running variable (Armstrong and Kolesár 2020) and a Donut RDiT excluding cohorts at the cut‐off. Secondary designs were an interrupted time series with a level shift and slope change at the famine cohort, and a within‐country region‐of‐birth × famine‐intensity difference‐in‐differences in the two Chinese cohorts that record province of birth—CHARLS (1959–1961 Great Famine) and CLHLS (1942–1943 Henan famine)—comparing famine‐exposed cohorts across high‐ versus low‐mortality provinces of birth (Chen and Zhou 2007); a two‐way fixed‐effects specification was reported. Three negative controls (a placebo cohort cut‐off, a placebo treatment window at ages 11–15 and a negative‐control outcome [biological sex]) probed bias. As sensitivity analyses, we report the reduced‐form association before and after adjustment for current age (a cubic polynomial), to quantify the age confounding; a leave‐one‐out analysis omitting each cohort, in particular the mortality‐selected Mexican cohort; and stratification by famine duration (≤ 2 vs. > 2 years). Risk differences (percentage points; pp) are reported for the design‐based analyses; the reduced‐form is reported as a risk ratio (Poisson). To assess whether the null‐design‐based estimates might instead reflect limited statistical power—the characteristic precision cost of confounding‐robust natural‐experiment designs (Craig et al. 2017)—we derived the minimum detectable effect at 80% power for each pooled design from its standard error, corroborated the primary RDiT with a 2000‐replication Monte‐Carlo power simulation, assessed equivalence to the null using two one‐sided tests and evaluated the power of the interrupted time series following the simulation‐based framework of Zhang et al. (2011), accounting for the autocorrelation of the series (Table S6; Figure S1). Analyses were performed in R 4.6.0 (rdrobust, RDHonest, rddensity, fixest, sandwich).

## 3 Results

Across the 165,586 participants in the analytical sample, famine‐exposed participants were older at survey than the post‐famine comparison cohort in every study, and unadjusted edentulism prevalence was correspondingly higher (Tables 1 and S1). The crude reduced‐form regression produced a positive association with edentulism (pooled RR 1.69, 95% CI: 1.55–1.84), but this was eliminated by adjustment for current age (RR 1.07, 95% CI: 0.92–1.23; *p* = 0.38; Table S2), and the famine‐exposed cohort did not differ from the older pre‐famine cohort (RR 0.97, 95% CI: 0.91–1.04; *p* = 0.42), indicating that the crude association reflects the cohort age gradient.

The quasi‐experimental designs were uniformly null. The primary pooled RDiT estimate was +0.13 pp. (95% CI: −1.50 to +1.77; *p* = 0.87), with a wider honest confidence interval (RDHonest −1.24 pp., 95% CI: −4.76 to +2.27) and non‐significant study‐specific estimates in all 10 cohorts (Figure 1). The interrupted time series showed no level shift at the famine cohort cut‐off (−4.01 pp.; 95% CI: −9.5 to +1.5; *p* = 0.15) against a smooth secular birth‐year trend (Figure 2). In the two Chinese cohorts, the region‐of‐birth × famine‐intensity difference‐in‐differences was null without covariate adjustment (CHARLS +0.15 pp., 95% CI: −1.45 to +1.75, *p* = 0.85; CLHLS +2.10 pp., 95% CI: −0.18 to +4.39, *p* = 0.07) and after adjustment (CHARLS −0.02 pp.; CLHLS +2.15 pp.; Figure 3). All three negative controls were null (placebo cohort +0.39 pp., *p* = 0.80; placebo window −2.14 pp., *p* = 0.47; negative‐control outcome −0.88 pp., *p* = 0.50). Leave‐one‐out analyses (including omission of the Mexican cohort) and stratification by famine duration did not change these conclusions (Tables S3 and S4).

**FIGURE 1:**
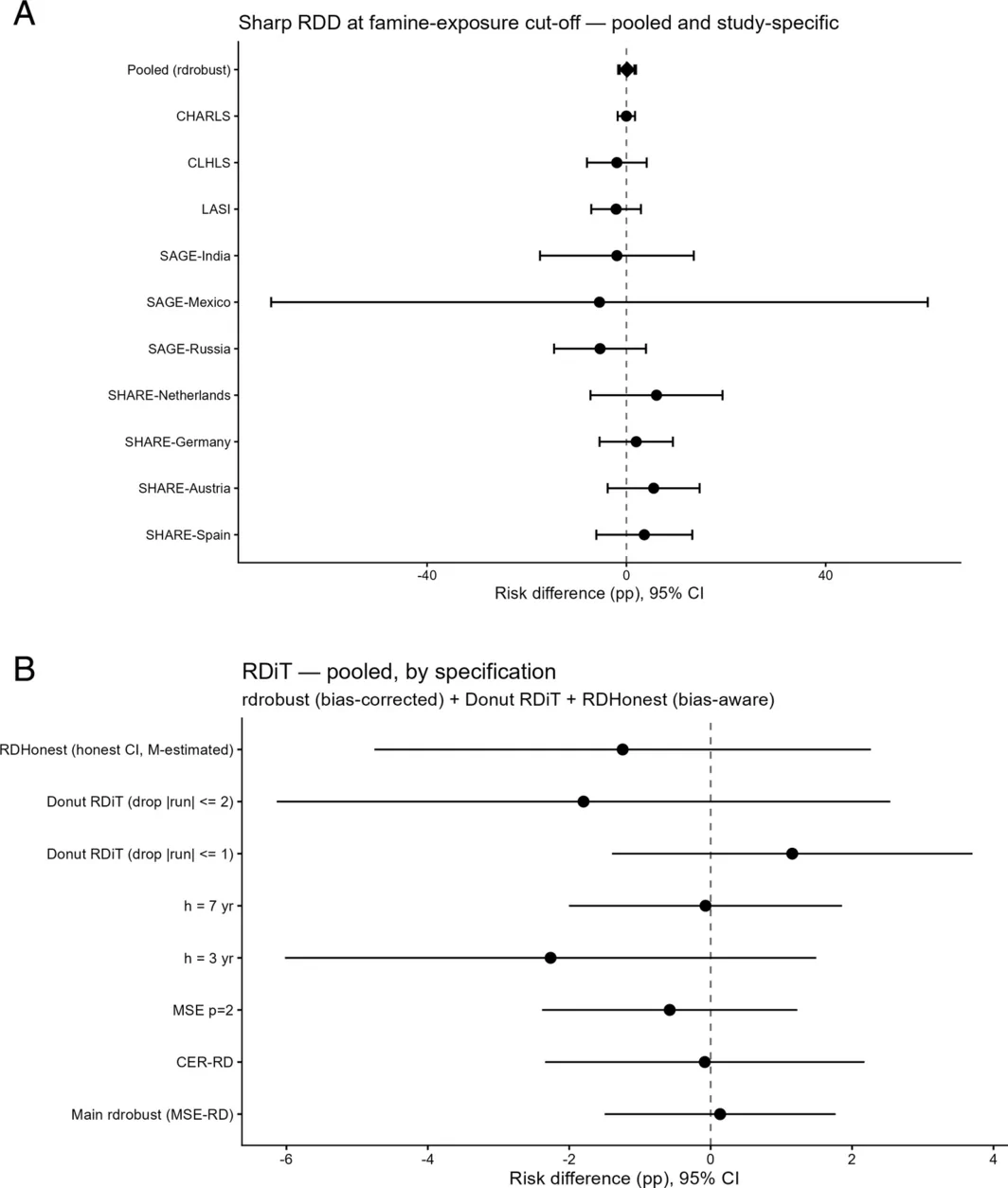
Regression discontinuity in time (RDiT) estimates of the effect of childhood famine exposure on late‐life edentulism (primary analysis). (A) Study‐specific RDiT estimates. (B) *Pooled RDiT specifications, including the bias‐aware honest CI*. Running variable: birth year minus the famine cohort cut‐off. Analytic sample 165,586 participants from 10 cohorts. Bias‐corrected, MSE‐optimal bandwidth, triangular kernel; honest bias‐aware CI for the discrete running variable; Donut RDiT excluding the cut‐off cohorts.

**FIGURE 2:**
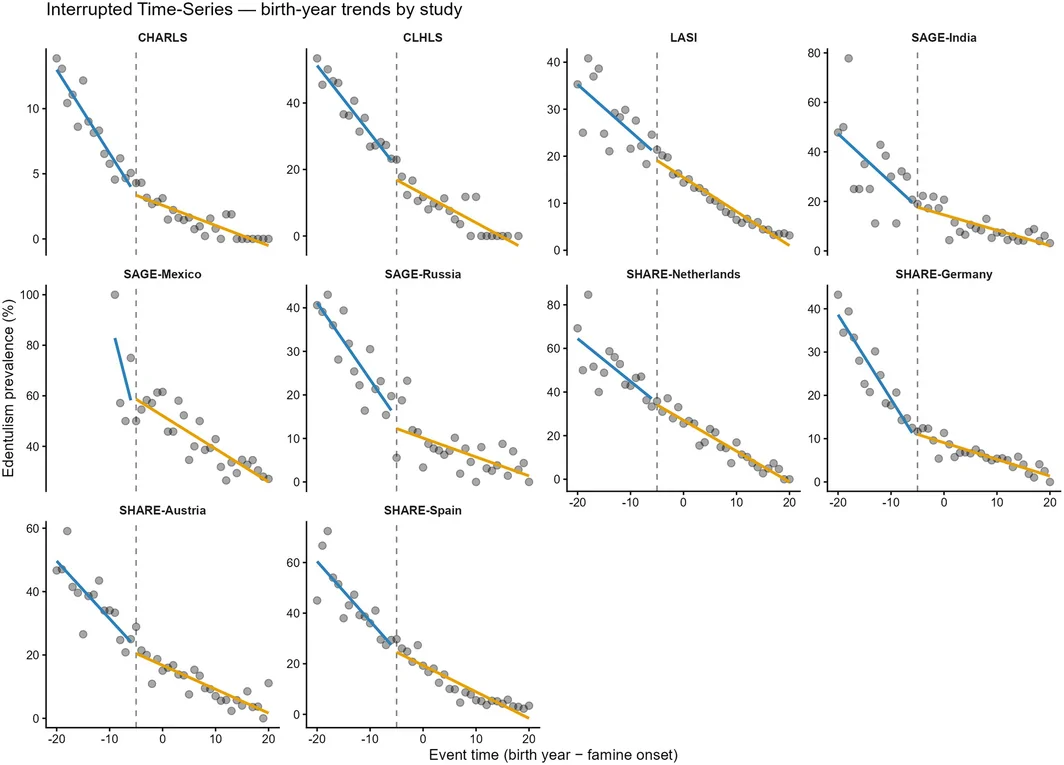
Interrupted time series of edentulism prevalence by birth year, pooled and by study (secondary analysis). Segmented regression with study fixed effects, weighted by cell size, robust standard errors. Vertical line: Famine cohort cut‐off.

**FIGURE 3:**
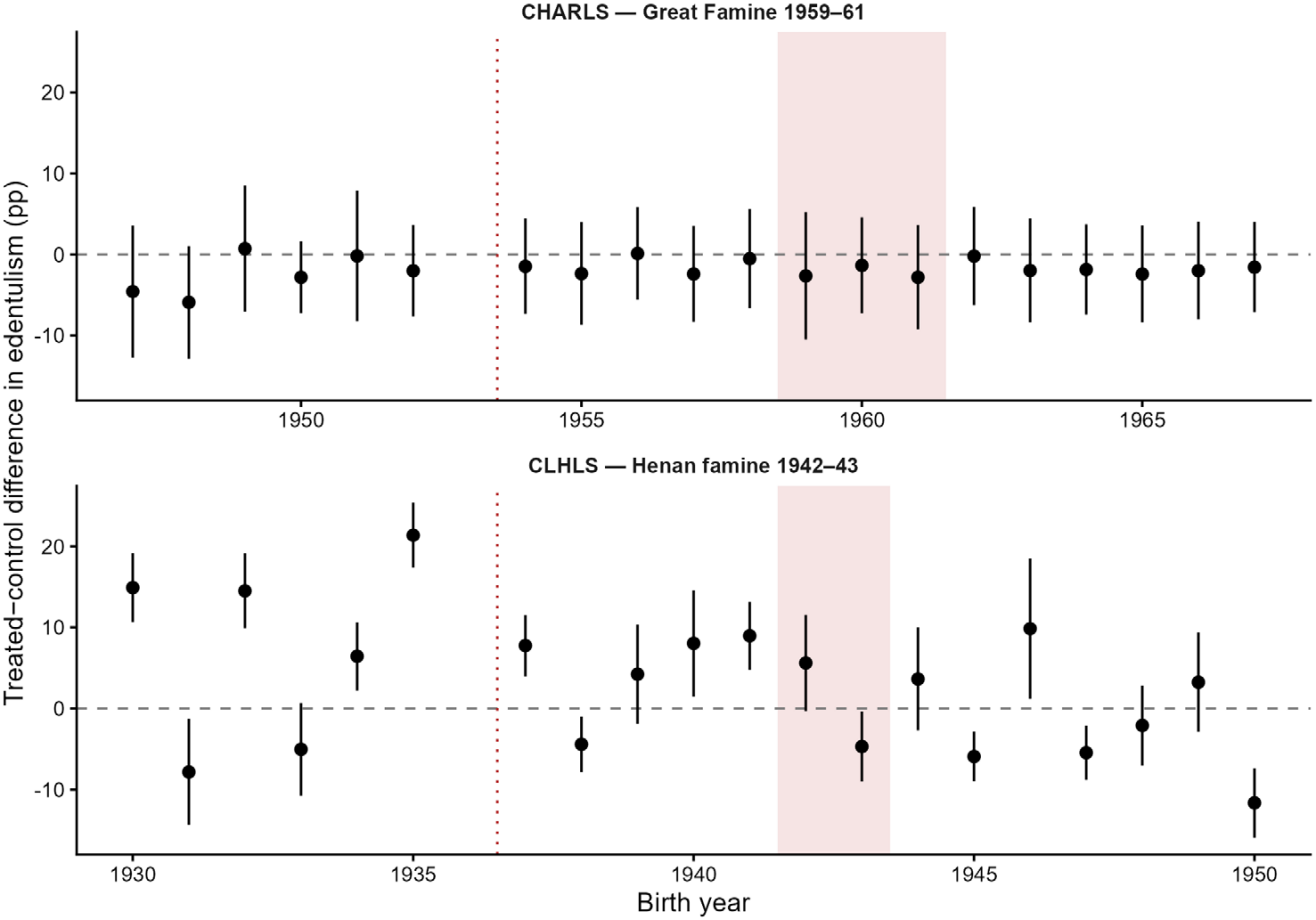
Within‐country region‐of‐birth × famine‐intensity difference‐in‐differences in CHARLS and CLHLS (secondary analysis). Childhood exposure is located by province of birth. Each point is the treated–comparison difference in edentulism by birth cohort, comparing high‐ versus low‐mortality provinces of birth (Chen and Zhou 2007). Dotted line: Birth‐year cutoff for early‐childhood famine exposure (children born from this year were aged ≤ 5 during the famine); shaded band: The famine years themselves (CHARLS 1959–1961; CLHLS 1942–1943).

## 4 Discussion

In this harmonised analysis of 10 famines spanning four continents and 165,586 older adults, multiple quasi‐experimental designs converged on a null result: childhood famine exposure showed no detectable effect on late‐life edentulism beyond approximately 2 pp. The reduced‐form RR fell from 1.69 to 1.07 after age adjustment; the famine cohort did not differ from the older pre‐famine cohort, and the primary regression‐discontinuity design, the interrupted time series and the region‐of‐birth difference‐in‐differences were all null, as were three pre‐specified negative controls. This also reconciles our findings with the positive cross‐sectional odds ratios reported by Gu et al. (2026): that analysis adjusts for age, but because its birth‐year‐defined cohorts occupy near‐non‐overlapping age bands, covariate adjustment incompletely separates age from cohort and leaves residual age confounding, which our age‐orthogonal designs remove.

The null finding is striking given that several biological pathways predict an effect. A plausible mechanism linking early‐life protein‐energy and micronutrient deficiency to periodontal disease operates through impaired neutrophil and T‐cell function, altered mucosal immunity and disrupted alveolar bone metabolism, leading to greater periodontal susceptibility. Furthermore, consistent with the DOHaD hypothesis, early famine exposure is linked to an increased risk of late‐life diabetes—an established risk factor for periodontitis. Together, these factors suggested a biological pathway: malnutrition → immune/metabolic dysfunction → periodontal breakdown → tooth loss (Alwarawrah et al. 2018; Bourke et al. 2016; Dommisch et al. 2018; Hujoel and Lingström 2017).

A second, less frequently acknowledged pathway, however, operates in the opposite direction. Historically, research on wartime diet and oral health has yielded conflicting narratives: studies from Austria, Ireland, Italy, Japan, Norway and the United Kingdom analysing children born during World Wars I and II reported that the prevalence of dental caries actually decreased precipitously during and shortly after wartime, an improvement largely associated with the severe reduction in sugar supplies during that time (Friel 1922; Massler and Schour 1947; Sognnaes 1948; Takeuchi 1961; Toverud 1957). This competing pathway is directly relevant here: in several of our populations (notably the Dutch Hongerwinter and the post‐war German and Austrian famines), wartime sugar restriction would have reduced caries‐related tooth loss, acting in the opposite direction to any harm from undernutrition.

Our null estimates indicate that at the population level, any famine‐induced increase in periodontitis may be masked by competing factors, most notably the reduction in dental caries resulting from wartime sugar restrictions. Because our harmonised edentulism outcome measure aggregates tooth loss driven by caries, periodontitis, trauma and prosthetic decisions, it represents a net effect, and the current data do not permit a periodontitis‐specific decomposition—meaning a true, pathway‐specific effect of childhood malnutrition on later life periodontal health cannot be entirely ruled out. In other words, the famine‐cohort design identifies a composite effect that bundles caloric restriction with non‐nutritional disruptions (psychological trauma, displacement, infectious disease, disrupted dental services); it can separate the net cohort effect from age and regional confounding but cannot isolate individual nutritional pathways.

This study has potential limitations. First, truncation by death (survivor bias) may bias the famine‐cohort comparison: the most vulnerable exposed individuals likely died before the survey, leaving a more robust survivor cohort that may also possess inherent genetic or epigenetic resilience to inflammatory conditions, including periodontitis, thereby biasing estimates towards the null (Gørgens et al. 2012). This selection is most extreme for the Mexican cohort, which has by far the longest interval between the famine and survey; a leave‐one‐out analysis omitting Mexico did not change the null conclusion. Second, the region‐of‐birth difference‐in‐differences was feasible only in the two Chinese cohorts, which record the province of birth. Third, the famine‐cohort exposure is composite, and the design cannot separate caloric restriction from co‐occurring non‐nutritional disruptions. Fourth, edentulism was self‐reported; given the retrospective recall, differential measurement error is possible. Fifth, natural experiment designs trade confounding control for statistical power (Craig et al. 2017); the secondary designs—the interrupted time series in particular (Zhang et al. 2011)—are the least powered, whereas the pooled analyses, above all the primary regression discontinuity, are well powered to detect effects of the magnitude previously reported (Gu et al. 2026) and make a large type‐II error unlikely (Table S6; Figure S1). Lastly, sample sizes were too small to examine effect modification by sex or place.

In conclusion, after separating famine exposure from the birth‐cohort age gradient using regression discontinuity, interrupted time series, and difference‐in‐differences designs, we find no consistent evidence that childhood famine exposure increases late‐life edentulism; the crude positive association is largely explained by age confounding. Cross‐sectional famine‐cohort comparisons that do not separate exposure from the age gradient may overstate early‐life effects on irreversible age‐dependent oral‐health outcomes (Li and Lumey 2017, 2022). This does not exclude pathway‐specific effects that may be counterbalanced by competing factors such as reduced caries from wartime sugar restriction; better evidence from younger, less mortality selected cohorts with clinical oral examinations is needed.

## Author Contributions

S.E.B. contributed to the conception, design, data analysis and interpretation, and drafted and critically revised the manuscript. C.M., T.K., and G.G.N. contributed to the conception, design and data interpretation, and critically revised the manuscript. All authors gave final approval and agreed to be accountable for all aspects of the work.

## Funding

The authors have nothing to report.

## Ethics Statement

Study ethical approval and data access CHARLS was accorded by the Biomedical Ethics Review Committee of Peking University (IRB00001052‐11015) https://charls.charlsdata.com/pages/data/111/en.html CLHLS Research Ethics Committee of Peking University (IRB00001052‐13074) https://opendata.pku.edu.cn/dataset.xhtml?persistentId doi:10.18170/DVN/WBO7LK LASI Indian Council of Medical Research (ICMR) and Institutional Review Boards (IRB) at IIPS and Harvard T.H. Chan School of Public Health https://g2aging.org/lasi/download SAGE WHO Ethical Review Committee (RPC 146) and National Ethical Review Committees in each participating country https://www.icpsr.umich.edu/web/ICPSR/studies/31381 SHARE Ethics Committee of the University of Mannheim (Waves 1–3); Ethics Council of the Max Planck Society (Waves 4+; Decisions dated 2010, 2012, 2014, 2018, 2020) https://share-eric.eu/data/data-access (Table S5).

## Consent

All participants provided informed consent.

## Supporting information


**Table S1:** Descriptive statistics by study and birth cohort, for the 165,586 participants in the analytic sample ṇpre‐famine = born more than 5 years before famine onset; famine‐exposed = born from 5 years before onset through the final famine year, that is, aged ≤ 5 years during the famine; post‐famine = born after the famine.
**Table S2:** Pooled reduced‐form association (famine‐exposed vs. immediately adjacent post‐famine cohort) before and after adjustment for current age. Poisson model with study fixed effects, risk ratios (RR); *n* is the number of participants contributing to each model.
**Table S3:** Leave‐one‐out sensitivity analysis (age‐adjusted reduced form, risk differences), omitting each cohort in turn.
**Table S4:** Stratification by famine duration (≤ 2 vs. > 2 years; age‐adjusted reduced form).
**Table S5:** Ethics approval and data access for the 10 included surveys.
**Table S6:** Statistical power and minimum detectable effect (MDE) of the regression discontinuity in time, reduced‐form, region‐of‐birth difference‐in‐differences and interrupted time series analyses.
**Figure S1:** Statistical power of the primary pooled regression discontinuity in time (RDiT). Monte‐Carlo power (blue points; 2000 replications per point; local‐linear RD with a triangular kernel at the MSE‐optimal bandwidth of 9.5 years) and the analytical power curve implied by the design's bias‐corrected standard error (orange), as a function of a true discontinuity injected at the famine cohort cut‐off. The dashed horizontal line marks 80% power and the dotted vertical line the corresponding minimum detectable effect of 2.3% points; the value at 0 pp. is the empirical type‐I error rate.

## Data Availability

The analysis code reproducing all tables and figures is openly available at https://github.com/BaumeisterS/famine-edentulism.
