# Supplementary material for: Childhood Famine Exposure and Late‐Life Edentulism: Evidence From Natural Experiments
Source: J Clin Periodontol. 2026 Aug 16;53(11):1673–80. doi: 10.1111/jcpe.70190 (PMC13658044; doi:10.1111/jcpe.70190)
Supplement: Supplementary file 1 [file JCPE-53-1673-s001.docx]

**Supplementary Materials**

**Contents**

Supplementary Table 1. Descriptive statistics by study and cohort.

Supplementary Table 2. Reduced-form association before and after age adjustment.

Supplementary Table 3. Leave-one-out sensitivity analysis.

Supplementary Table 4. Stratification by famine duration.

Supplementary Table 5. Ethics approval and data access.

Supplementary Table 6. Statistical power and minimum detectable effect (MDE) of the regression discontinuity in time, reduced-form, region-of-birth difference-in-differences and interrupted time-series analyses.

Supplementary Figure 1. Statistical power of the primary pooled regression discontinuity in time (RDiT).

**Supplementary Table 1.** Descriptive statistics by study and birth cohort, for the 165,586 participants in the analytic sample (pre-famine = born more than five years before famine onset; famine-exposed = born from five years before onset through the final famine year, i.e. aged ≤5 during the famine; post-famine = born after the famine).

| **Study** | **Cohort** | **n** | **Age, mean (SD)** | **Birth year** | **% male** | **% edentulous** |
| --- | --- | --- | --- | --- | --- | --- |
| CHARLS | Pre-famine | 7599 | 65.4 (6.6) | 1945 | 51.2 | 9.6 |
| CHARLS | Famine-exposed | 4598 | 53.6 (2.3) | 1957 | 49 | 3.2 |
| CHARLS | Post-famine | 4849 | 46.1 (2.9) | 1965 | 42.6 | 1.2 |
| CLHLS | Pre-famine | 14632 | 90 (9.5) | 1918 | 40.7 | 59 |
| CLHLS | Famine-exposed | 1991 | 67.7 (2.1) | 1940 | 53.6 | 14.5 |
| CLHLS | Post-famine | 282 | 58.9 (3.6) | 1949 | 70.6 | 6.4 |
| LASI | Pre-famine | 2940 | 85.3 (5) | 1932 | 47.8 | 26.7 |
| LASI | Famine-exposed | 4919 | 76.2 (2.1) | 1941 | 49.5 | 17.2 |
| LASI | Post-famine | 65320 | 55.3 (9.3) | 1962 | 41.6 | 4.6 |
| SAGE-India | Pre-famine | 559 | 73.6 (6.6) | 1929 | 57.2 | 33.6 |
| SAGE-India | Famine-exposed | 758 | 62.1 (2.3) | 1941 | 48.2 | 19.5 |
| SAGE-India | Post-famine | 8273 | 34.4 (10.9) | 1969 | 47.9 | 3.5 |
| SAGE-Mexico | Pre-famine | 20 | 96.7 (1.1) | 1906 | 25 | 70 |
| SAGE-Mexico | Famine-exposed | 157 | 88.9 (2.3) | 1914 | 43.9 | 54.8 |
| SAGE-Mexico | Post-famine | 23982 | 41 (16.6) | 1962 | 41.8 | 7.6 |
| SAGE-Russia | Pre-famine | 1416 | 72.6 (6.4) | 1930 | 28.1 | 35.5 |
| SAGE-Russia | Famine-exposed | 380 | 58.9 (2.2) | 1944 | 36.1 | 10.8 |
| SAGE-Russia | Post-famine | 2570 | 38.4 (10.5) | 1965 | 40.5 | 3.6 |
| SHARE-Netherlands | Pre-famine | 855 | 80.3 (5) | 1932 | 47.3 | 48.3 |
| SHARE-Netherlands | Famine-exposed | 799 | 70.2 (2.1) | 1942 | 47.7 | 30.7 |
| SHARE-Netherlands | Post-famine | 2461 | 59.1 (5.2) | 1953 | 42.9 | 13.2 |
| SHARE-Germany | Pre-famine | 1233 | 78.8 (4.8) | 1933 | 49.2 | 22.1 |
| SHARE-Germany | Famine-exposed | 1403 | 68.2 (2.8) | 1944 | 52 | 9.1 |
| SHARE-Germany | Post-famine | 3046 | 56 (4.9) | 1956 | 43.5 | 4.6 |
| SHARE-Austria | Pre-famine | 1078 | 79.7 (5.2) | 1933 | 41.3 | 36.5 |
| SHARE-Austria | Famine-exposed | 1322 | 68.6 (2.8) | 1944 | 44 | 18.3 |
| SHARE-Austria | Post-famine | 1807 | 57.4 (4.6) | 1955 | 41.4 | 8.1 |
| SHARE-Spain | Pre-famine | 1151 | 84.3 (4.2) | 1928 | 42.9 | 38.7 |
| SHARE-Spain | Famine-exposed | 1347 | 74.6 (2.7) | 1938 | 48 | 21.7 |
| SHARE-Spain | Post-famine | 3839 | 59.9 (6.1) | 1953 | 45.7 | 5.6 |

CHARLS = China Health and Retirement Longitudinal Study; CLHLS = Chinese Longitudinal Healthy Longevity Survey; LASI = Longitudinal Aging Study in India; SAGE = Study on Global Ageing and Adult Health; SHARE = Survey of Health, Ageing and Retirement in Europe

**Supplementary Table 2.** Pooled reduced-form association (famine-exposed vs immediately adjacent post-famine cohort) before and after adjustment for current age. Poisson model with study fixed effects, risk ratios (RR); n is the number of participants contributing to each model.

| **Model** | **RR** | **95% CI** | **p** | **n** |
| --- | --- | --- | --- | --- |
| Unadjusted | 1.69 | 1.55–1.84 | <0.001 | 25,753 |
| + sex + early-life SES | 1.68 | 1.54–1.83 | <0.001 | 25,753 |
| + cubic current age | 1.07 | 0.92–1.23 | 0.38 | 23,697 |

**Supplementary Table 3.** Leave-one-out sensitivity analysis (age-adjusted reduced form, risk differences), omitting each cohort in turn.

| **Study omitted** | **Estimate (pp)** | **95% CI** | **p** |
| --- | --- | --- | --- |
| CHARLS | +1.33 | (-0.78, +3.45) | 0.22 |
| CLHLS | +1.12 | (-0.57, +2.80) | 0.19 |
| LASI | +1.09 | (-0.72, +2.90) | 0.24 |
| SAGE-India | +0.24 | (-1.40, +1.88) | 0.78 |
| SAGE-Mexico | +0.74 | (-0.88, +2.36) | 0.37 |
| SAGE-Russia | +0.84 | (-0.80, +2.47) | 0.32 |
| SHARE-Netherlands | +0.34 | (-1.27, +1.96) | 0.68 |
| SHARE-Germany | +1.01 | (-0.69, +2.71) | 0.24 |
| SHARE-Austria | +0.94 | (-0.71, +2.60) | 0.26 |
| SHARE-Spain | +0.81 | (-0.85, +2.47) | 0.34 |

**Supplementary Table 4.** Stratification by famine duration (≤2 vs >2 years; age-adjusted reduced form).

| **Famine duration** | **Estimate (pp)** | **95% CI** | **p** | **n** |
| --- | --- | --- | --- | --- |
| Short | +2.68 | (-0.39, +5.74) | 0.087 | 8469 |
| Long | -0.62 | (-2.47, +1.23) | 0.512 | 15228 |

**Supplementary Table 5.** Ethics approval and data access for the ten included surveys.

| **Study** | **Ethical approval** | **Data access** |
| --- | --- | --- |
| CHARLS | Biomedical Ethics Review Committee of Peking University (IRB00001052-11015) | https://charls.charlsdata.com/pages/data/111/en.html |
| CLHLS | Research Ethics Committee of Peking University (IRB00001052-13074) | ttps://opendata.pku.edu.cn/dataset.xhtml?persistentId=doi:10.18170/DVN/WBO7LK |
| LASI | Indian Council of Medical Research (ICMR) and Institutional Review Boards (IRB) at IIPS and Harvard T.H. Chan School of Public Health | https://g2aging.org/lasi/download |
| SAGE | WHO Ethical Review Committee (RPC 146) and national ethical review committees in each participating country | https://www.icpsr.umich.edu/web/ICPSR/studies/31381 |
| SHARE | Ethics Committee of the University of Mannheim (Waves 1-3); Ethics Council of the Max Planck Society (Waves 4+; Decisions dated 2010, 2012, 2014, 2018, 2020) | https://share-eric.eu/data/data-access |

All participants provided informed consent at data collection; no additional approval was required for the pooled secondary analysis.

**Supplementary Table 6.** Statistical power and minimum detectable effect (MDE) of the regression discontinuity in time, reduced-form, region-of-birth difference-in-differences and interrupted time-series analyses.

| **Design** | **SE (pp)** | **MDE, 80% power (pp)** | **MDE, 90% power (pp)** |
| --- | --- | --- | --- |
| Regression discontinuity in time — conventional | 0.83 | 2.3 | 2.7 |
| Regression discontinuity in time — robust (bias-corrected) | 1.00 | 2.8 | 3.2 |
| Regression discontinuity in time — honest (RDHonest) | 1.79 | 5.0 | 5.8 |
| Reduced-form risk difference (age-adjusted) | 0.82 | 2.3 | 2.7 |
| Region-of-birth difference-in-differences — CHARLS | 0.82 | 2.3 | 2.7 |
| Region-of-birth difference-in-differences — CLHLS | 1.17 | 3.3 | 3.8 |
| Interrupted time-series (level shift) | 2.78 | 7.8 | 9.0 |

Minimum detectable effect (MDE) at 80% and 90% power for a two-sided test at α = 0.05, derived from each design’s estimated standard error (SE). On the risk-ratio scale the age-adjusted reduced form had 80% power to detect a risk ratio of 1.20. For an effect of the magnitude reported by Gu et al. (2025) (adjusted odds ratios of 2–4, equivalent to risk differences of 8–18 percentage points at the 8.6% post-famine baseline prevalence of edentulism), power exceeded 99% for the primary RDiT and the reduced form. The primary RDiT was statistically equivalent to zero within a ±2 percentage-point margin (two one-sided tests, p = 0.01). The interrupted time-series was the least-powered design; accounting for the first-order autocorrelation of the birth-year series (ρ = 0.46) by generalised least squares, following the simulation-based framework of Zhang et al. (2011), widens its standard error to 3.6 pp (minimum detectable level shift ≈10 pp). A 2,000-replication Monte-Carlo simulation of the primary RDiT corroborated these values (Supplementary Figure 1).

**Supplementary Figure 1.** Statistical power of the primary pooled regression discontinuity in time (RDiT).

Monte-Carlo power (blue points; 2,000 replications per point; local-linear RD with a triangular kernel at the MSE-optimal bandwidth of 9.5 years) and the analytic power curve implied by the design’s bias-corrected standard error (orange), as a function of a true discontinuity injected at the famine cohort cut-off. The dashed horizontal line marks 80% power and the dotted vertical line the corresponding minimum detectable effect of 2.3 percentage points; the value at 0 pp is the empirical type-I error rate.
